# Wild type p53 function in p53^Y220C^ mutant harboring cells by treatment with Ashwagandha derived anticancer withanolides: bioinformatics and experimental evidence

**DOI:** 10.1186/s13046-019-1099-x

**Published:** 2019-02-26

**Authors:** Durai Sundar, Yue Yu, Shashank P. Katiyar, Jayarani F. Putri, Jaspreet Kaur Dhanjal, Jia Wang, Anissa Nofita Sari, Evangelos Kolettas, Sunil C. Kaul, Renu Wadhwa

**Affiliations:** 1 DAILAB, Department of Biochemical Engineering & Biotechnology, Indian Institute of Technology (IIT) Delhi, Hauz Khas, New Delhi, 110 016 India; 2 DAILAB, DBT-AIST International Center for Translational and Environmental Research (DAICENTER), National Institute of Advanced Industrial Science & Technology (AIST), Central 5, 1-1-1 Higashi, Tsukuba, Ibaraki, 305 8565 Japan; 3 Laboratory of Biology, School of Medicine, Faculty of Health Sciences, University of Ioannina, and Biomedical Research Division, Institute of Molecular Biology and Biotechnology, Foundation for Research and Technology, 45110 Ioannina, Greece

**Keywords:** Withaferin A, Withanone, p53 mutants, Wild type p53 restoration, Cancer therapy

## Abstract

**Background:**

Tumor suppressor p53 protein is frequently mutated in a large majority of cancers. These mutations induce local or global changes in protein structure thereby affecting its binding to DNA. The structural differences between the wild type and mutant p53 thus provide an opportunity to selectively target mutated p53 harboring cancer cells. Restoration of wild type p53 activity in mutants using small molecules that can revert the structural changes have been considered for cancer therapeutics.

**Methods:**

We used bioinformatics and molecular docking tools to investigate the structural changes between the wild type and mutant p53 proteins (p53^V143A^, p53^R249S^, p53^R273H^ and p53^Y220C^) and explored the therapeutic potential of Withaferin A and Withanone for restoration of wild type p53 function in cancer cells. Cancer cells harboring the specific mutant p53 proteins were used for molecular assays to determine the mutant or wild type p53 functions.

**Results:**

We found that p53^V143A^ mutation does not show any significant structural changes and was also refractory to the binding of withanolides. p53^R249S^ mutation critically disturbed the H-bond network and destabilized the DNA binding site. However, withanolides did not show any selective binding to either this mutant or other similar variants. p53^Y220C^ mutation created a cavity near the site of mutation with local loss of hydrophobicity and water network, leading to functionally inactive conformation. Mutated structure could accommodate withanolides suggesting their conformational selectivity to target p53^Y220C^ mutant. Using human cell lines containing specific p53 mutant proteins, we demonstrated that Withaferin A, Withanone and the extract rich in these withanolides caused restoration of wild type p53 function in mutant p53^Y220C^ cells. This was associated with induction of p21^WAF-1^-mediated growth arrest/apoptosis.

**Conclusion:**

The study suggested that withanolides may serve as highly potent anticancer compounds for treatment of cancers harboring a p53^Y220C^ mutation.

**Electronic supplementary material:**

The online version of this article (10.1186/s13046-019-1099-x) contains supplementary material, which is available to authorized users.

## Introduction

p53 protein has been established as a tumor suppressor and guardian of the genome. It inhibits proliferation of genetically altered or stressed cells by induction of growth arrest, senescence or apoptosis [1]. It also blocks the metastasis and angiogenesis of cancer cells. In the absence of stress, wild type p53 (p53^WT^) undergoes rapid degradation, regulated by HDM2 and other negative regulators like Pirh2, COP1 and mortalin [2–5] accounting for its short half-life in normal cells. Besides, p53 regulates its own stability by structural modulation [6]. Under stressed conditions like genotoxic damage, oncogene activation or hypoxia, it is stabilized and activated by post-translational modifications [7, 8]. Activated p53 then either induces growth arrest or apoptosis in the dividing cells [9, 10] curtailing the proliferation of genetically stressed/damaged cells that carry high risk of carcinogenesis.

Inactivation of p53 protein is the key factor in uncontrolled proliferation of cells. Mutated p53 with altered function or complete inactivation has been detected in over 85% of cancers [11, 12]. Genetic changes in p53 results in (i) altered interactions with proteins like ubiquitin ligases leading to modified levels of ubiquitination [13], (ii) exclusion of p53 from nucleus [5], (iii) abrogation of p53-DNA interactions [14] or (iv) unstable tetramer structure, essential for p53 to function as a transcriptional activator [12]. More than 7500 single missense point mutations that affect the central core of p53, involved in direct binding with DNA have been reported (http://p53.iarc.fr/). The DNA binding domain (DBD) of p53 stretches from 112 to 286 amino acids (Additional file 1: Fig. S1). Mutations in the DBD either disrupt the DNA binding directly or bring local/global change in the p53 protein structure. It is comprised of immunoglobulin-like β-sandwich, made up of two antiparallel β-sheets facilitating an elastic DNA-binding surface [14]. Two large loops (L2 and L3) stabilized by a zinc ion and a loop–sheet–helix motif (loop L1) collectively make the DBD. Four residues His 179, Cys 176, Cys 238 and Cys 242 stabilize the zinc ion via coordinate bonds. Zinc binding is critical for the correct folding of p53 and requires the reduction of thiol group on cysteines. The first category of mutations involves changes in amino acid residues 175, 242, 248, 249, 273 and 282. These mutations trigger local changes around the DNA-binding site of the protein and prevent its binding to DNA. Amongst these, mutations at 175, 248 and 273 are most commonly found in cancer patients and known as hotspot mutations of p53 [15]. Furthermore, whereas alteration in Arg 248 and Arg 273 are described as ‘contact mutations’, alteration in Arg 175, Gly 245, Arg 249 and Arg 282 are classified as ‘structural mutations’ [16]. Although the overall structure of these mutant p53 proteins remain similar to the wild type protein, they still show loss of critical DNA contact residues. Introduction of residues with large hydrophobic side chain in DBD also prevents interaction with DNA as in case of S241F, R248W and C277F mutants of p53. This category also includes the mutants (e.g., C176F, H179R and C242F) that affect the zinc binding domain of p53. Histidine, due to its interference with zinc binding, causes structural distortions that mainly include the formation of internal cavities or surface crevices in the scaffold of p53, inducing conformational changes in the DNA binding surface. Studies on thermodynamic stability of these mutants have revealed that p53^WT^ and p53^R273H^ do not differ much in their stability, whereas p53^C242S^ and p53^R174H^ can be destabilized by 2.9 kcal/mol and 3.0 kcal/mol, respectively [17]. Similarly, p53^R248Q^ and p53^R249S^ are less stable in comparison to wild type and p53^R273H^ mutant [17]. The second class of p53 mutants cause global change in the structure of protein and hence disrupt the stability of p53. In contrast to the first category of mutations that directly disrupt the DNA-binding, these mutations are less frequent, occur in β-sandwich and mostly disturb hydrophobicity of the core region. So far, nine mutations have been reported to occur in this region - V143A (S3), L145Q (S3), P151S (S3-S4 loop), V157F (S4), I195T (S5), Y220C (S7-S8 loop), I232T (S8), I255F (S9) and F270C (S10) [6]. These p53 mutants, although retain their DNA-binding affinity, can be destabilized by 3.0 to 4.5 kcal/mol of energy. Although Pro 151, Val 157 and Tyr 220 are located far from the DNA-binding region, these are significant mutational hotspots within the beta-sheet of the protein. p53^V143A^ and p53^Y220C^ mutants have been reported to be temperature sensitive for transactivation under in vivo conditions [18]. These possess 68% of the wild type p53-DNA binding activity at 20 °C, however, get denatured at 37 °C leading to oncogenic outcomes.

The thermodynamic differences between the wild type and the mutant p53 suggest that it is a highly sensitive protein, especially to temperature. The free energy differences between the mutant and wild type are significantly small, paving way for the therapeutic interventions. Attempts have been made to rescue p53 function by stabilizing the mutant structures. Several reports have confirmed that the restoration of p53 function leads to tumor suppression and senescence [19, 20]. Several peptides and small molecule-based approaches have been devised to revert the mutant to wild type p53 function [21–23]. Some of these include treatment with mAb PAb421 [24], C-terminal peptide of p53 [25], peptide CDB3 [26] and small molecules such as CP-31398 [27], PRIMA1 [28], SCH529074 [29], PhiKan083 [30] and PK7088 [31]. Targeting mutant p53 protein for cancer treatment offers advantages like - (i) high selectivity to cancer cells, as mutant p53 is not present in normal cells, and (ii) designed peptides and small molecules can further offer specificity and high efficacy to a particular type of mutant p53.

Low doses of Withaferin A (Wi-A) and Withanone (Wi-N), withanolides present in Ashwagandha (*Withania somnifera*) leaves, have been reported to selectively kill a variety of cancer cells [32–36]. The cytotoxicity has been shown to be mediated by selective activation of wild type p53 and abrogation of cancer cell specific mortalin-p53 interactions [5, 34]. It was shown that Wi-N and Wi-A dock into the mortalin-p53 binding site preventing their interaction and thereby resulting in nuclear translocation and reactivation of p53 [4, 5, 36–38]. Furthermore, in our earlier study, it was indicated that Wi-N may restore wild type like p53 activity in cancer cells harboring mutant p53 [34]. In the present study, we undertook extensive bioinformatics and experimental analyses to investigate the binding of withanolides, Wi-A and Wi-N, with specific hot spot p53 mutants. Among the several mutants that were analyzed, we observed that these withanolides interacted with p53^Y220C^ mutant, but not the p53^WT^, exhibiting conformational selectivity for binding. By cell-based p53 assays, we provide experimental evidence to the restoration of wild type p53 function in p53^Y220C^ mutants. We thus provide the first line of evidence that Wi-A and Wi-N may be particularly effective for treatment of cancers with Y220C p53 mutation.

## Materials and methods

### Wild type and mutant p53 proteins

Structure of the wild type p53 (p53^WT^) and various mutants - V143A (p53^V143A^), Y220C in complex with a small molecule (p53^Y220C^), unbound Y220C (p53^Y220C^), R249S (p53^R249S^), R273C (p53^R273C^), and R273H (p53^R273H^) were downloaded from Protein Data Bank (Additional file 1: Table S1). Wild type and all the mutant protein structures contained the core domain of p53. These structures were prepared for docking studies using protein preparation wizard of Schrödinger software [39, 40]. The binding site in p53^Y220C^ was identified based on co-crystalized molecule, PhiKan in 3ZME. For the rest of the mutant proteins, the site of mutation was explored for the presence of cavities using SiteMap module of Schrodinger that were further used for the generation of grid for docking experiments. For p53^WT^, four grids were generated near residue number 143, 220, 249 and 273, respectively.

### Molecular docking studies

Structures of Wi-A and Wi-N were obtained from PubChem. These structures were pre-processed to get the clean structure with correct molecular mechanics parameters and atom types. The ligands were prepared for the docking experiments using LigPrep protocol of Schrodinger [41]. Withanolides were then docked at the generated grids of p53 protein structures using XP docking protocol of Glide [41].

### Molecular dynamics setup

The dynamic stability of the docked complexes was then studied using the approach of molecular dynamics (MD) simulations. GPU accelerated Amber molecular dynamics suite with Amber ff99SB/ff12SB protein force field was used to perform all atoms explicit MD simulations. Firstly, hydrogen atoms were added to each protein-ligand complex using Tleap module in AMBER 12 software package [42]. Antechamber program was used to generate force field parameters for the ligand as described by the general AMBER force fields (GAFF) [43]. The prepared complexes were solvated using TIP3P water model [44] in an octahedron box with boundary at approximately 10 Å distance around the protein. The solvated systems were then neutralized by adding appropriate number of counter ions, followed by minimization, heating and equilibration before the production MD simulation run. Minimization was carried out in two steps. Firstly, only the water molecules were minimized keeping force restraints over protein-ligand complexes, which was then followed by the minimization of the whole system. The first 3000 steps of energy minimization were carried out with steepest descent method and the remaining 2000 steps with conjugate gradient method. Particle Mesh Ewald (PME) summation was used to handle the long-range coulombic interactions with cut-off value of 10 Å. Minimized systems were then slowly heated to bring system’s temperature from 0 K to 300 K in NVT ensemble with time step of 0.005 fs. The systems were then equilibrated until pressure and density of systems were stabilized in NPT ensemble. For equilibration and subsequent steps, Berendsen thermostat was used in NPT ensemble with target pressure of 1 bar and pressure coupling constant of 2 ps. At the end, the production phase of MD was run using the same conditions for a duration of 30 ns for each complex. The SHAKE algorithm was turned on for all atoms covalently bonded to a hydrogen atom that allowed an integrative time step of 2 fs [45]. Minimization, heating and equilibration of protein-withanolide complexes were run using a small force restraint of 10 kcal/mol/Å^2^ over the docked withanolide.

### Cell culture

Human hepatocarcinoma HUH-6 and HUH-7 cells were purchased from the Japanese Collection of Research Bioresources (JCRB, Japan). The cells were authenticated by the source. Cells were frozen in -80 °C and LN_2_ in multiple vials and cultured (described below) for less than 50 population doublings for the present study. MRC-5 (non-transformed diploid human lung fibroblasts) and its derivatives; telomerised MRC-5 TERT fibroblasts [46] and the MRC-5 TERT/p53^V143A^ fibroblasts [47] have been described previously. Briefly, amphotropic phoenix cells (3 × 10^6^) were transfected with 10 μg of the retroviral plasmids using the calcium phosphate precipitation method and then MRC-5 were infected with either control Babe-Puro or Babe-Puro/hTERT retroviruses in the presence of 8 μg/ml polybrene, and then selected in 1 μg/ml puromycin selection for two weeks. Stable MRC-5 TERT cells were then infected with ΔH and ΔHp53^V143A^ packaged in Phoenix cells or with ZIP-Neo/p53^R273H^ and ZIP-Neo/p53^R249S^ [48] produced by PA317 cells and subjected to 50 μg/ml hygromycin or to 200 μg/ml G418, respectively, for three weeks. Cells were cultured in Dulbecco’s modified Eagle’s medium (DMEM; Gibco BRL, Grand Island, NY, USA) and treated with Wi-A at about 60% confluency. Morphological observations and cell viability (MTT and colony-forming assays) were determined as described in the following sections.

### Antibodies

Rabbit anti-PARP-1 (H-250), anti-caspase-3 (H-277), goat anti-PML (N-19) (Santa Cruz), mouse anti-p53 (DO-1 and Fl-393; pan-p53 antibodies recognizing wild type as well as mutant p53 epitopes) (sc-126, Santa Cruz) [49], Y5-detecting mutant p53 only (ab32049, Abcam); rabbit anti-PARP-9, anti-p21^WAF-1^ (12D1) (Cell Signaling Technology) antibodies were used. HP1γ (clone 4252), Phospho p53 (Ser-15) (clone 9284) and anti-53BP1 (BD Transduction Laboratories) were purchased from Millipore and Cell signaling, respectively.

### Cell viability assay

Cells were seeded into 96-well plate (10^4^ cell/well) for 24 h and treated with Wi-A for next 48 h. Cell viability was determined by MTT (3-(4,5-Dimethylthiazol-2-yl)-2,5-Diphenyltetrazolium Bromide) (Molecular Probes, Invitrogen) following the manufacturer’s instructions. Control and treated cells were incubated with MTT (0.5 mg/ml) at 37 °C, 5% CO_2_ for 4 h. MTT-containing medium was replaced with DMSO (100 μl). Absorbance of the blue chromogen was measured at 570 nm using a spectrophotometer (TECAN, Switzerland). The standard deviation and statistical significance of the data was obtained from triplicates and 3–4 independent experiments, respectively.

### Immunoblotting

Cells were cultured, treated as indicated (results section) and lysed using RIPA buffer containing protease inhibitor cocktail (Roche Applied Science, Mannheim, Germany). Protein was quantified using Bicinchonic Acid Assay (BCA) (Thermo Fisher Scientific, Rockford, IL). The cell lysate (15–20 μg) was resolved on a SDS-PAGE and transferred to polyvinylidene difluoride (PVDF) membrane (Millipore, Billerica, MA). Membrane containing proteins was incubated with specific antibodies at 4 °C overnight. Membrane was incubated with secondary antibodies conjugated with horseradish peroxidase anti-rabbit IgG and anti-mouse IgG (Cell signaling technology) and developed by enhanced chemiluminescence reaction (ECL) (Elpis Biotech, Daejeon, Korea).

### Immunostaining

Cells were seeded on glass coverslips placed into 12-well plate overnight and treated with withanolides a day later as described earlier. Cells were fixed with methanol:acetone [1], washed with PBS, permeabilized with 0.1% Triton X-100 in PBS, and blocked with 2% BSA. Fixed cells were incubated with antibodies (as indicated) at 4 °C overnight followed by incubation with Alexa Flour conjugated antibodies at room temperature for 45 mins. Counterstaining was performed using Hoechst 33342 (Sigma) for 5 min and mounted. Stained cells were examined on a Zeiss Axiovert 200 M microscope and analyzed by AxioVision 4.6 software (Carl Zeiss). At least, 200 cells, on duplicate slides, were evaluated for each treatment condition for co-localization or foci counting. Three independent experiments were performed. Images were quantified by ImageJ software (National Institute of Health, Bethesda, MD).

### Comet assay

DNA Comet Assay was used to detect DNA-Double Strand Breaks following the instructions from Comet Assay® (Trevigen, MD USA) protocols.

### Apoptosis and cell cycle analyses

Apoptosis and growth arrest detection were examined following the manufacturer’s instructions from Guava Nexin® and Guava Cell Cycle® Reagents (Millipore, Ma USA). Quantification of data was done using FlowJo 7.6 software.

### Luciferase reporter assay

Plasmid WWP-luc carrying the full p21^WAF-1^ promoter was purchased from AddGene, MA USA. PG13-Luc bearing 13 repeats of the p53^WT^ binding sequence was a kind gift from Professor Bert Vogelstein. Plasmids were transfected into cells using Lipofectamine 2000 (Invitrogen, Carlsbad CA, USA). Luciferase activity was detected using the Dual-Luciferase® Reporter Assay System (Promega, WI, USA) following the manufacturer’s instructions.

### Statistical analysis

The data from three or more experiments were expressed as mean ± standard deviation (SD). Two-tailed Student’s t-test was used to compare control and treated groups. Statistical significance was defined as significant (*p-value ≤0.05), very significant (**p-value ≤0.01) and highly significant (***p-value ≤0.001).

## Results

### Docking of Wi-A and Wi-N to p53 wild type (WT) and mutant (V143A, R249S, R273C and R273H) proteins

Mutations at four amino acid residues (175, 248, 249 and 273) account for over 25% of all p53 missense mutations identified in human cancers, and have been linked to various characteristics of cancer cells [11, 50, 51]. Whereas the R273H mutation disrupts the DNA damage response leading to genetic instability and tumorigenesis, V143A has been shown to promote cancer metastasis. p53^R249S^ and p53^Y220C^ mutations have been shown to be the major mutations in Aflatoxin B1-related hepatocellular carcinoma and head & neck squamous cell carcinoma [52]. Of note, the p53^Y220C^ mutation has been categorized as the 9^th^ most frequent p53 missense mutation found in cancer with ~ 100,000 new cancer cases per year worldwide [53]. In these premises, we investigated the structural differences between the wild type and these mutant proteins and the ability of Wi-A and Wi-N to interact with wild type/mutant p53 structures.

**Fig. 1:**
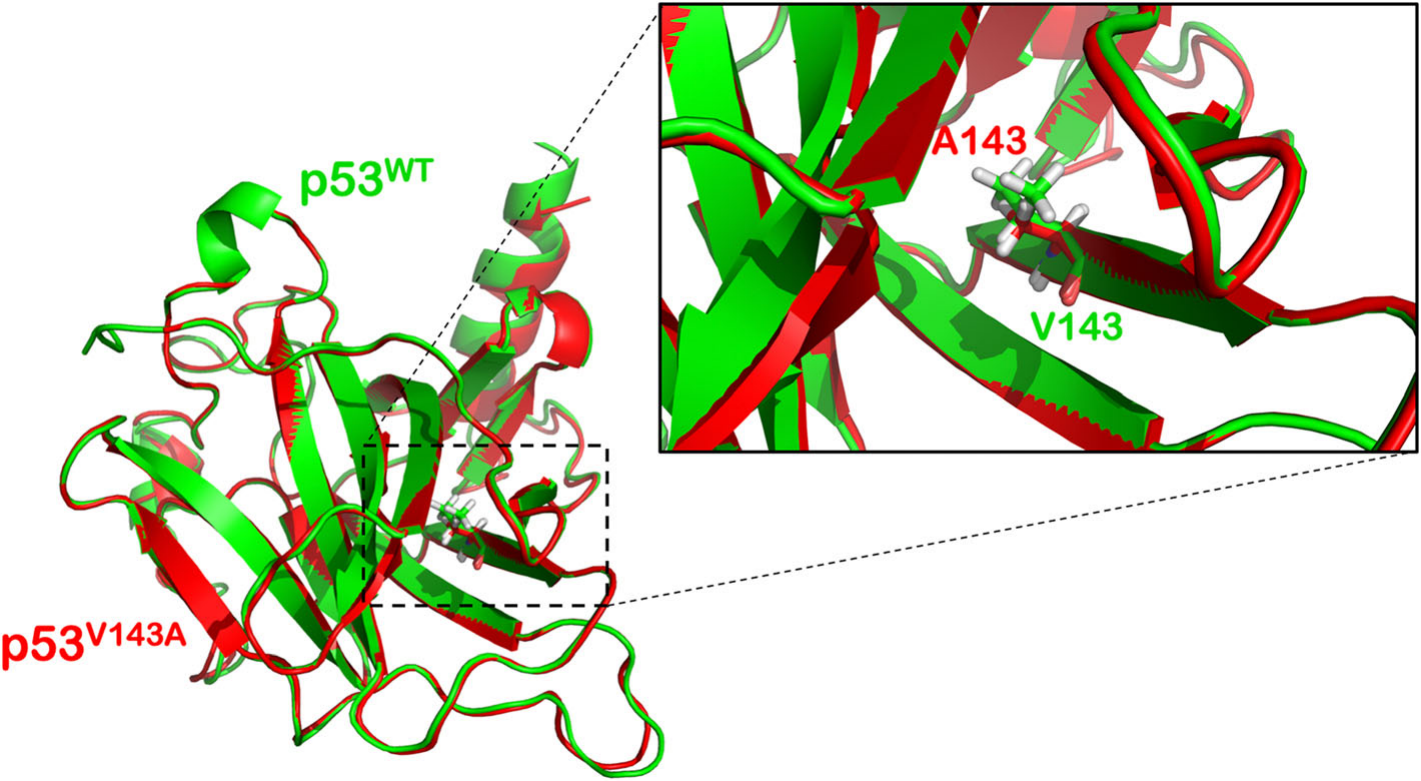
Structural comparison between p53^WT^ and p53^V143A^. Superimposed structures of p53^WT^ (green) and p53^V143A^ (red) are shown. The mutation did not affect the local structure or DNA binding site

**Fig. 2:**
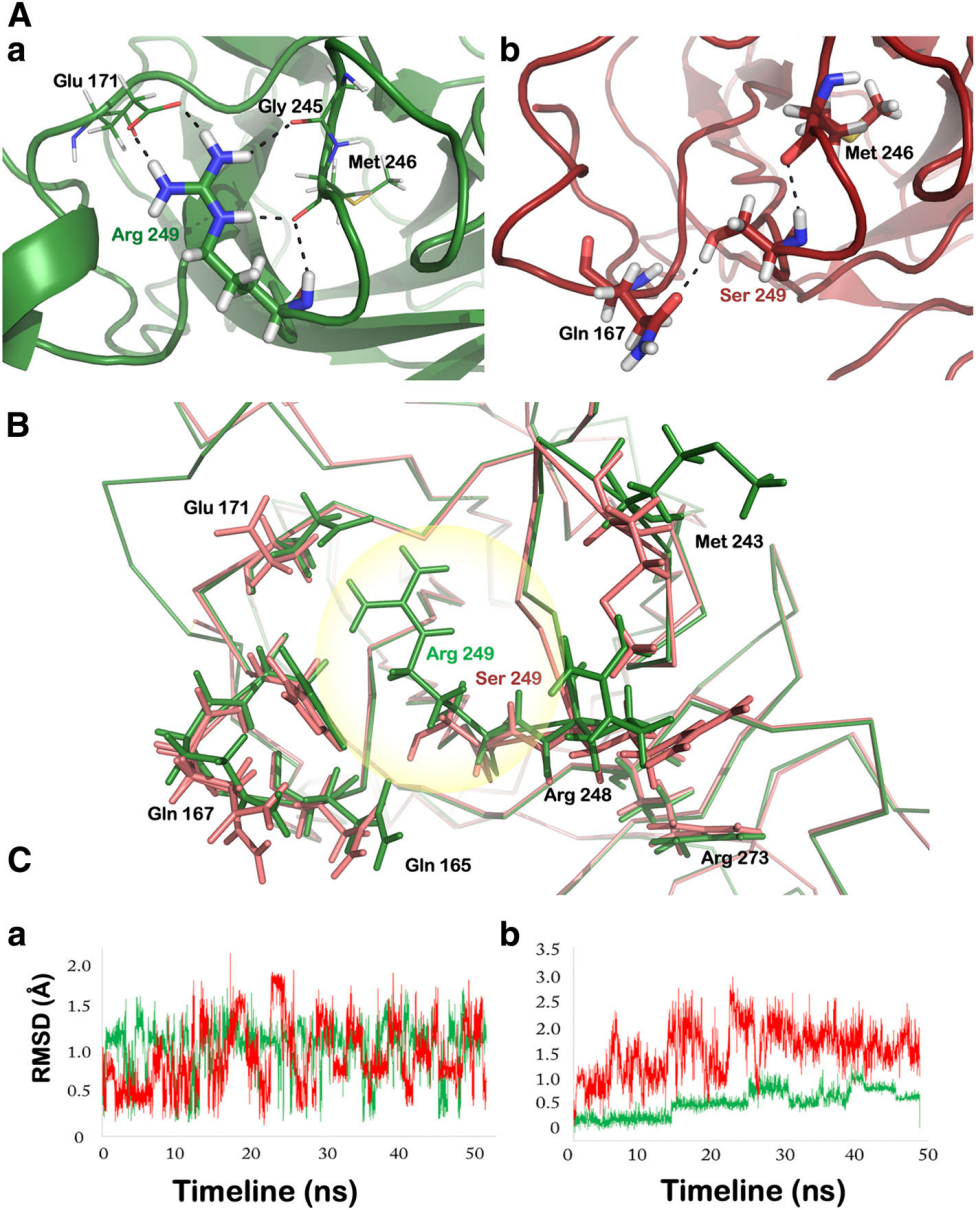
**a** Polar interactions formed by Arg 249 with neighbouring residues in p53^WT^. **b** Change in polar contacts with neighbouring residues due to mutation of Arg 249 to serine in p53^R249S^. **b** Superimposed view of the mutation site in p53^WT^ and p53^R249S^. Residues of p53^WT^ are shown in green and for p53^R249S^ they are shown in pink. Change in the structure at 249th position is highlighted by a circle. Residues that acquired significantly different conformations are shown in stick representation. **c** Root mean square deviations of residues in p53^WT^ and p53^R249S^. (a) RMSD of Arg 248 during the course of MD simulation in p53^WT^ (green) and in p53^R249S^ (red). (b) RMSD of residues from 249 to 271 in p53^WT^ (green) and p53^R249S^ (red)

The comparison between p53^WT^ and p53^V143A^ revealed no structural differences around the site of mutation (Fig. 1). The overall protein structural root mean square deviation (RMSD) between p53^WT^ and p53^V143A^ was found to be 0.67 Å. Superimposition of the two proteins showed good alignment of the secondary structures, even around the site of mutation, reflecting no structural alterations due to the presence of mutated residue. Furthermore, Wi-N/Wi-A did not show binding near the target amino acid residue (143 position) either in wild type or p53^V143A^. The R249S mutation in p53 was found to be destabilizing in nature with an increase in the solvent accessible surface area. Although this amino acid does not directly interact with the DNA, the mutation disrupted the binding of p53 with DNA by inducing unfavorable structural changes in its DNA binding region (Fig. 2). Thermodynamically, this mutation led to destabilized p53 structure by 1.9 kcal/mol [6]. In order to investigate further, we examined the local hydrogen bond network in p53^WT^ and p53^R249S^ around the site of mutation. As shown in Fig. 2a (a), Arg 249 formed five hydrogen bonds involving three p53 residues - Gly 245, Met 246 and Glu 171. In contrast, in p53^R249S^, most of these hydrogen bonds were lost (Fig. 2a (b)). A detailed H-bond network analysis is shown in Additional file 1: Table S2. Considering the local region harboring the mutation, the total number of the H-bonds reduced from twelve to nine. Conformational changes were also observed in residues surrounding Ser 249 that mainly included Gln 165, Gln 167, Glu 171, Arg 248 and His 168 (Fig. 2b). To further investigate the effect of mutation on the structure of p53, both p53^WT^ and p53^R249S^ were subjected to 50 ns MD simulations. Along with the movement of Arg 248 (Fig. 2c (a)), a major effect was observed on the region ranging from 249 to 271 amino acids leading to an unstable DNA binding region in p53^R249S^ (Fig. 2c (b)). Wi-A and Wi-N were docked with both wild type and mutant protein near the location of change in amino acid. However, neither of the two withanolides showed any selectivity against the p53^R249S^ mutant. The binding score of Wi-A with p53^WT^ was − 4.09 kcal/mol that did not change with the mutant protein as well. Similarly, Wi-N showed binding score of − 3.71 kcal/mol with p53^WT^ and − 3.95 kcal/mol with p53^R249S^. Since no selective effect was observed for Wi-N or Wi-A against p53^R249S^, this p53 variant was not studied further. A similar analysis was carried out for other variants of p53 (p53^R273C^ and p53^R273H^), where the mutated residues were found in the DBD of p53. Withanolides were unable to selectively distinguish these two p53 variants from the wild type p53 (Additional file 1: Table S3) and hence were also not studied further.

### Structural analysis of p53^Y220C^

**Fig. 3:**
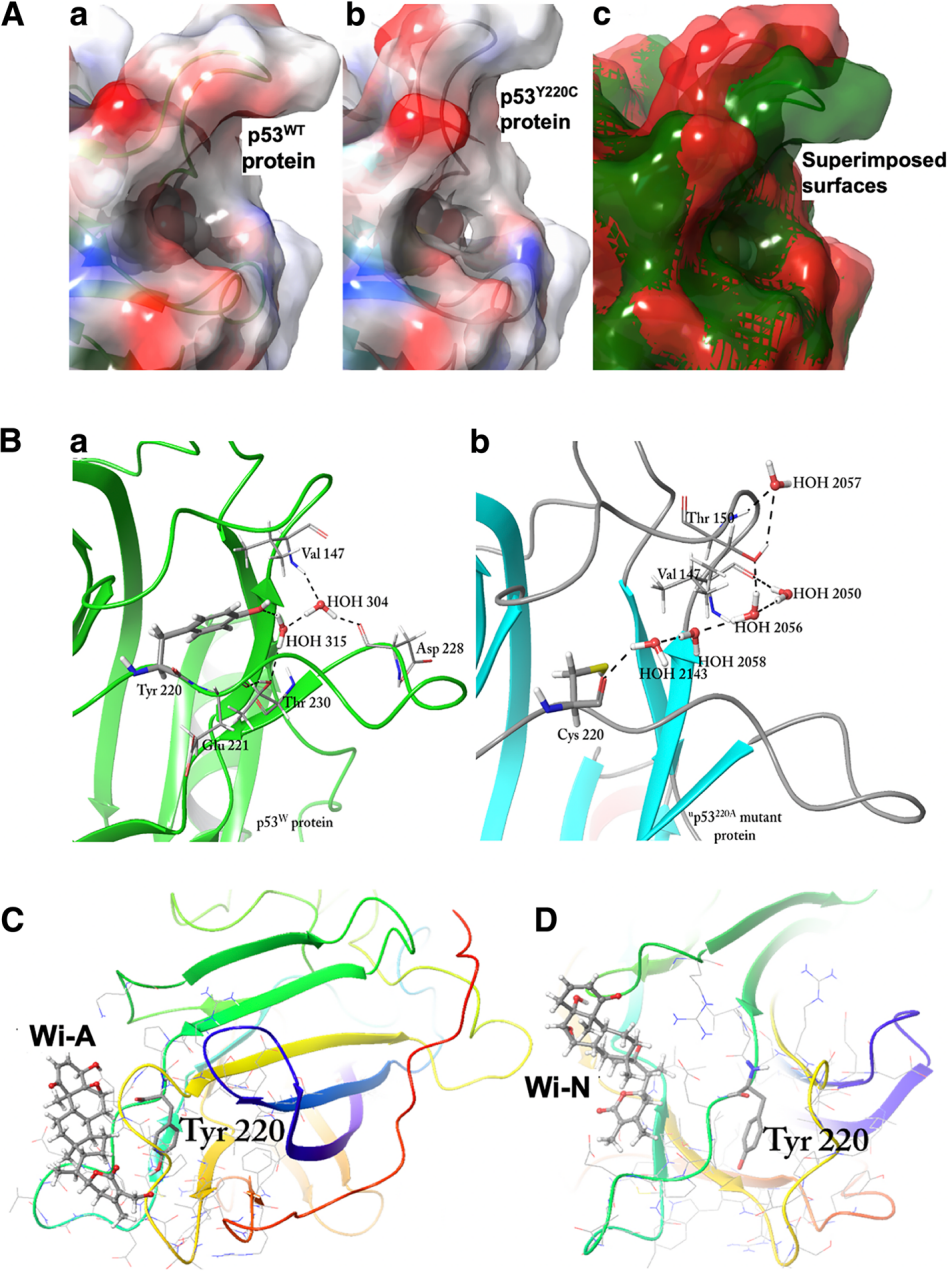
**a** Difference in the molecular surface near Y220 position in p53^WT^ and p53^Y220C^ mutant. a Size of cavity in p53^WT^. b Mutation from Tyr to Cys at 220th position rearranges the conformation of surrounding residues, especially located in the loop region. Widening of the loop near 220th position and removal of the Tyr side chain creates a larger cavity in p53^Y220C^ protein. c Superimposition of the two molecular surfaces reveals the stringency of the p53^WT^ (green color) as compared to the p53^Y220C^ (red color). **b** Water network near 220th residue in p53^WT^ and p53^Y220C^ protein. a Tyr 220 stabilizes the residues of surrounding loops with the help of water molecules. b Cys 220 stabilizes the cavity by solvating it with water molecules. Binding of the withanolides with p53^WT^ near Tyr 220. Wi-A **c** and Wi-N **d** were found to interact with the surface residues near Tyr 220 as no deep cavity was present in p53^WT^

Structural analysis of p53^Y220C^ revealed the existence of a deeper and wider cavity around the location of mutation in comparison to the wild type protein (Fig. 3a). This newly formed cavity was a result of linkage between two pre-existing shallow cavities in p53^WT^ [54]. Despite these changes in molecular surface near the site of mutation, no global change occurred in Y220C mutant with respect to the wild type protein. To further explore the role of tyrosine, its surrounding hydrogen bond network was analyzed. It was found to stabilize the nearby loop by the formation of hydrogen bonds via stable water molecules surrounding it. A close look at the PDB structure 3KMD showed a hydrogen bond between Tyr 220 and water HOH 315 that in turn was forming a hydrogen bond with Thr 230 and another water molecule, HOH 304. HOH 304 was further found to be interacting with Val 147 and Asp 228 (Fig. 3b (a)). Analysis of p53^Y220C^ structure (PDB ID: 2J1X) showed the presence of water molecules - HOH 2050, HOH 2056, HOH 2057, HOH 2058 and HOH 2143 near the site of mutation (Fig. 3b (b)).

### Binding of withanolides with p53^Y220C^ near Tyr 220

**Fig. 4:**
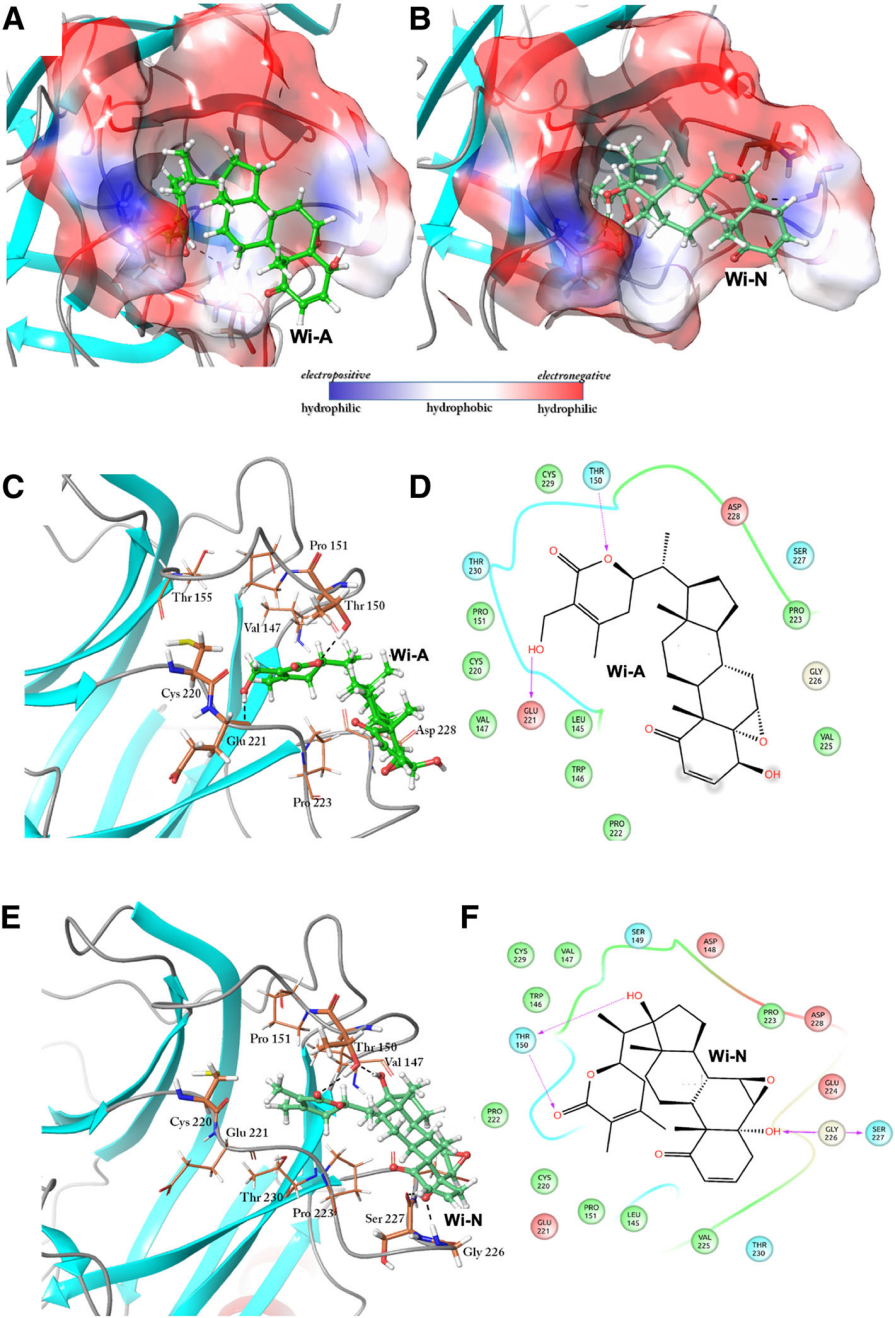
Interactions of Wi-A with p53^Y220C^ near Cys 220. **a** Binding pose of Wi-A within the binding site near Cys 220 **b** 2D representation of the interactions of Wi-A with p53^Y220C^. Interactions of Wi-N with p53^Y220C^ protein structure. **c** Binding pose of Wi-N within the binding cavity of p53^Y220C^ near Cys 220 **d** 2D representation of the interactions of Wi-N with surrounding residues within the cavity of p53^Y220C^. Charge complementarity of Wi-A and Wi-N with the binding cavity. Wi-A **e** and Wi-N **f** both were fitting inside the cavity according to the charge distribution within it. White region represents the hydrophobic region whereas blue and red represents the hydrophilic region

We next considered the information available on PhiKan7242 and PhiKan083, small molecules known to have the ability of reversing the effect of Y220C mutation in p53 [50–57]. PhiKan molecules are available as co-crystallized ligands only with mutant p53 in PDB. We first generated docking scores of PhiKan molecules in their bound position in p53^Y220C^ mutant. To get an estimate of their interaction with p53^WT^ and p53^Y220C^, both the PhiKans and withanolides (Wi-N and Wi-A) were docked with these proteins around Tyr 220, also the documented site of interaction for Phikans with p53^Y220C^. Because of a shallow cavity in p53^WT^, Phikans and withanolides failed to dock with high docking score (Additional file 1: Table S4). Withanolides were found to be randomly interacting with some residues located on the exposed surface near Tyr 220 (Fig. 3c and d) and therefore the binding was not significant. However, on the other hand, binding of PhiKans and withanolides with p53^Y220C^ near the site of mutation yielded profound docking poses with high glide scores. The docking score for Wi-A, Wi-N, PhiKan7242, and PhiKan083 was − 3.42 kcal/mol, − 4.3 kcal/mol, − 3.65 kcal/mol and − 4.76 kcal/mol, respectively. Further analysis was carried out to compare the interaction pattern of withanolides with that of PhiKans along with the exploration of structural changes incurred due to these interactions. Wi-A and Wi-N were observed to bind near Cys 220 within the extended cavity of the mutant p53 as in case of PhiKans. The lactone ring of Wi-A was found to be embedded within the cavity, whereas the steroidal part with ketone, hydroxyl and epoxy hydrophilic groups was exposed to the solvent (Fig. 4a). A similar binding orientation was adopted by Wi-N as well (Fig. 4b). The details of molecular interactions of both the withanolides with p53^Y220C^ are shown in Fig. 4c-f.

### Wi-A furnished wild type p53 function in p53^Y220C^ mutant harboring cancer cells - experimental evidence

**Fig. 5:**
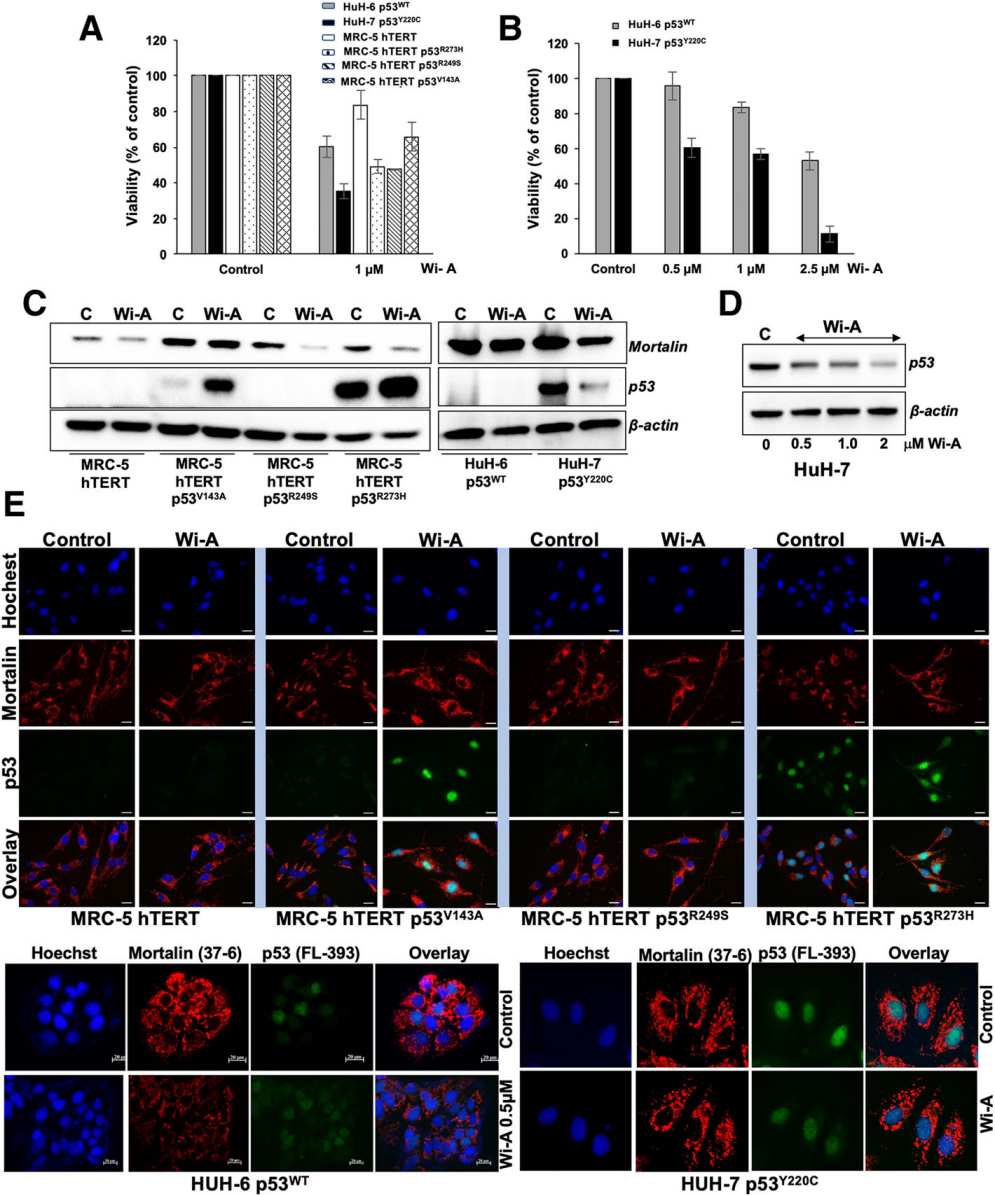
Wi-A furnished wild type p53 function in mutant p53 (p53^Y220C^) horboring hepatoma cells. **a** Viability assay of human hepatocarcinoma with wild type p53 (HuH-6), mutant p53 (HuH-7), and telomerized human cells bearing p53 mutants (p53^V143A^, p53^R249S^ and p53^R273H^). **b** Comparison of response of HuH-6 and HuH-7 cells to Wi-A. Dose response was observed for both the cell lines. HuH-7 showed stronger cytotoxicity to Wi-A. **c** Western blot showed reduction in mortalin and increase in p53 in cells treated with 1 μM Wi-A in the p53 mutants, p53^V143A^ and p53^R273H^; p53^R249S^ cells possessed low expression that remained undetected on these blots. In contrast to increase in the expression of p53^V143A^ and p53^R273H^, mutant p53^Y220C^ protein expression was decreased in Wi-A treated cells. **d** Dose dependent decrease in mutant p53^Y220C^ protein expression in Wi-A treated cells. **e** Immunostaining of mortalin and p53 (40 x magnification) in control and Wi-A (0.5 μM) showing increase in nuclear p53^V143A^ and p53^R273H^. HuH-6 (p53^WT^) cells showed increase in nuclear p53 staining. In contrast, HuH-7 (p53^Y220C^) cells exhibited decrease in p53 nuclear staining

**Fig. 6:**
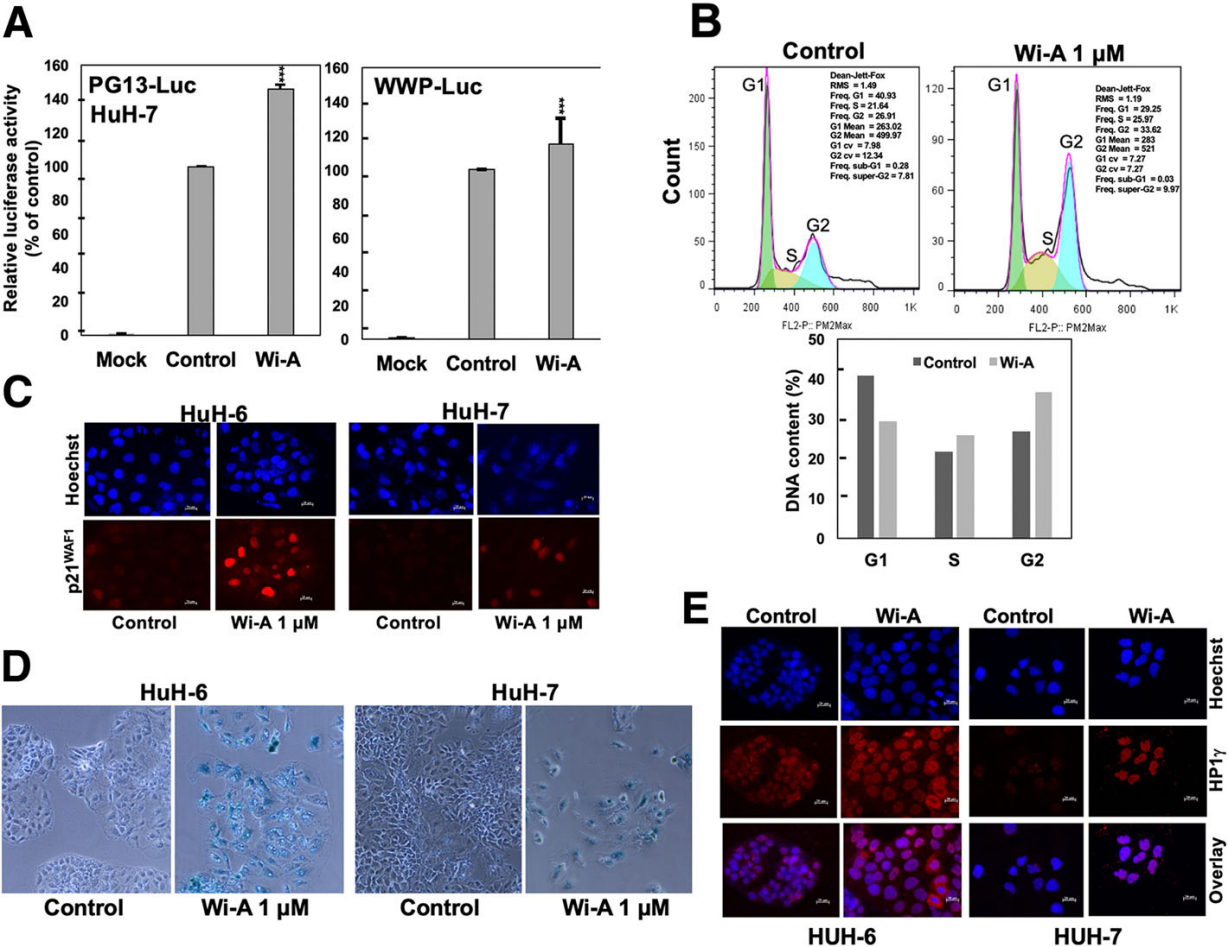
Wi-A induced restoration of wild type p53 and induction of senescence in HuH-7 cells. **a** Wild type p53 reporter activity in mock (untransfected), control (transfected and untreated) and Wi-A (transfected and Wi-A treated) cells. Luciferase reporter assays driven either by p53 consensus binding sites (PG13-Luc) or by a p21^WAF-1^ promoter (WWP) showed an increase in Wi-A treated cells. **b** Flow cytometry analysis revealed G_2_ cell cycle phase arrest in HuH-7 cells. **c** Immunostaining of p21^WAF-1^ in HuH-6 and HuH-7 control and Wi-A treated cells showing increase in p21^WAF-1^ expression in the latter. **d** Senescence-associated β-galactosidase staining was observed in Wi-A treated HuH-6 and HuH-7 cells (10 x phase magnification). **e** Wi-A treated HuH-6 and HuH-7 cells showed enhanced staining for nuclear heterochromatin protein HP1γ

Next, we experimentally studied the above predictions by investigating the effect of Wi-A on cells expressing the above p53 mutant proteins. As shown in Fig. 5a and data not shown, all cell lines responded to 0.5 to 1 μM Wi-A. While the low doses of Wi-A caused growth arrest, high doses triggered apoptosis [34] (data not shown). Of note, hepatocarcinoma HuH-7 cells expressing p53^Y220C^ were most sensitive to Wi-A treatment. Dose response experiment confirmed that Wi-A caused higher toxicity to cells harboring p53^Y220C^ mutant as compared to the wild type p53 expressing HuH-6 cells (Fig. 5b). The status of p53 in control and treated cells was confirmed by Western blotting with antibodies that reacted to pan or specifically mutant p53 protein. As shown in Fig. 5c, there was an increase in p53^V143A^ and p53^R273H^ mutant protein expression in Wi-A treated cells compared to their untreated counterparts. As expected, MRC-5/hTERT and HuH-6 cells showed a low level of expression due to its short half-life [57]. p53^R249S^ also showed a low level of expression whereas high level of mutant p53^V143A^ and p53^R273H^ expression was detected and showed further increase in Wi-A treated cells. In contrast, p53^Y220C^ mutant protein showed clear decrease in Wi-A treated cells as compared to the control cells (Fig. 5c). In keeping with this, we showed a dose dependent decrease in p53^Y220C^ mutant protein expression in cells treated with increasing doses of Wi-A (Fig. 5d). Immunostaining using pan and mutant p53 protein specific antibodies (Fig. 5e and Additional file 1: Figure S2) further confirmed these results. As shown in Fig. 5e, Wi-A caused an increase in nuclear translocation of p53^V143A^ and p53^R273H^ mutants. p53^R249S^ was not detected and p53^Y220C^ showed a clear decrease. Furthermore, immunostaining of mortalin exhibited shift in its staining pattern from perinuclear (typical of cancer cells) to pancytoplasmic (typical of normal cells) suggesting abgoration of mortalin-p53 complexes in Wi-A treated cells, as has also been demonstrated earlier [33–35]. Importantly, whereas an increase in nuclear p53 was observed in Wi-A treated p53^V143A^ and p53^R273H^ cells, a decrease was observed for p53^Y220C^ (Fig. 5e, Additional file 1: Figure S4A and B). These data indicated restoration of wild type characteristics (rapid degradation) to p53^Y220C^. In order to examine this further, we performed wild type p53-dependent luciferase reporter assays in control and Wi-A treated cells using two reporters, a p53 binding consensus sequence (PG13-Luc) and a p21 promoter (WWP-Luc). As shown in Fig. 6a, Wi-A caused an increase in wild type p53 activity in mutant p53 harboring HuH-7 cells endorsing the wild type transcriptional activation function of p53. Consistent with the activated wild type p53 function, the cells exhibited a cell cycle arrest prominently at G_2_ phase (Fig. 6b) that was correlated with (i) increase in p21^WAF-1^ (Fig. 6c and Additional file 1: Figure S4C) and (ii) induction of senescence as determined by senescence-associated β-gal expression (Fig. 6d) as well as HP1γ staining (Fig. 6e and Additional file 1: Figure S4D) in HUH-7 cells. HuH-6 (p53^WT^) cells, used as control, were induced to senescence like growth arrest by Wi-A [34, 36] and showed similar activation of wild type p53 function. As shown in Figs. 6c-e, control and Wi-A treated HuH-7 cells showed induction of p21^WAF-1^ and cellular senescence, marked by senescence-associated β-gal staining (Fig. 6d), heterochromatin foci (HP1γ) (Fig. 6e) and cell morphology (enlarged flattened cells, data not shown). These data confirmed the restoration of wild type p53 activity in HuH-7 cells, similar to HuH-6 cells bearing p53^WT^. Furthermore, in addition to the flat senescent cells, we found apoptotic cells in Wi-A treated HuH-7 cells, especially at higher (1–2 μM) doses.

**Fig. 7:**
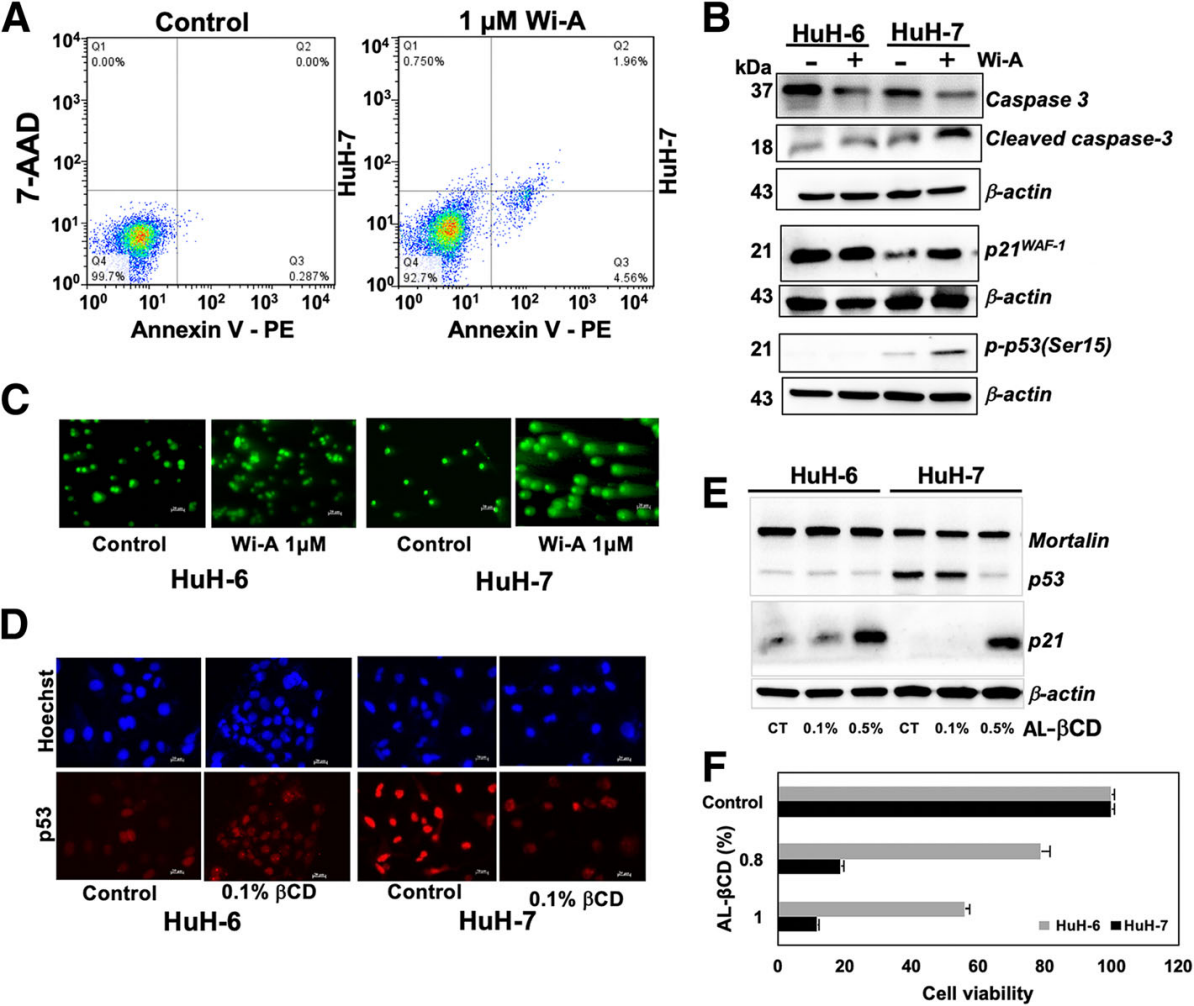
Wi-A induced apoptosis of HuH-7 cells. **a** Annexin-V staining revealed induction of early apoptosis in Wi-A treated cells. **b** Apoptosis in Wi-A treated HuH-7 cells was marked by cleavage of caspase3, increase in p21^WAF-1^ and phosphorylated p53. **c** Wi-A treated cells showed Single Strand Breaks as determined by a comet assay, 40 X magnification. **d** Wi-A rich extract (AL-βCD) treated HuH-6 (p53^WT^) and HuH-7 (p53^Y220C^) cells showed increase and decrease in nuclear p53, respectively. **e** Increase in the expression of p21^WAF-1^ was observed in AL-βCD treated HuH-6 and HuH-7 cells. **f** HuH-7 cells showed strong cytotoxicity to AL-βCD

We next determined if the induction of wild type p53 activity in p53^Y220C^ cells could also induce apoptosis. Annexin V staining, as determined by flow cytometery analysis, showed significant increase in apoptotic cells in Wi-A treated cultures (24–48 h) as compared to their untreated counterparts (Fig. 7a). Wi-A induced apoptosis in HuH-7 cells was also confirmed by cleavage of caspase 3 (Fig. 7b). An increase in p21^WAF-1^ expression was confirmed by immunoblotting (Fig. 7b) in addition to immunostaining (Fig. 6c). Furthermore, phosphorylation of p53 at Ser 15 that plays a critical role in transactivation function of p53^WT^ [58] was detected (Fig. 7b) confirming the wild type p53 function in Wi-A treated HuH-7 cells. We next examined DNA damage response in HuH-7 cells. Immunostaining for 53BP1 and p21^WAF-1^ in control and Doxorubicin (an established DNA damage inducing drug) treated cells revealed increase in 53BP1 and p21^WAF-1^ in HuH-7, similar to HuH-6 cells, implying that they are responsive to DNA damage (Additional file 1: Figure S3) resulting in growth arrest. We next examined whether reconstitution of wild type p53 function in HuH-7 cells by Wi-A would also result in activation of DNA damage response. As shown in Fig. 7c, Comet Assay showed remarkable DNA damage response in Wi-A treated cells.

In view of the above data, we hypothesized that Ashwagandha leaf extracts (AL) enriched in Wi-A and Wi-N could be valuable for restoration of wild type p53 function in p53^Y220C^ harboring cells. As described earlier [59], β-CD extracts that possess high content of withanolides, especially Wi-A and Wi-N were tested. HuH-6 and HuH-7 cells were treated with AL-βCD extracts. As shown in Fig. 7d and Additional file 1: Figure S4D, while HuH-6 showed mild increase in p53^WT^ level of expression, a decrease in mutant p53^Y220C^ in HuH-7 cells was observed. Furthermore, increase in p21^WAF-1^ occurred in both HuH-6 and HuH-7 cells (Fig. 7e) signifying wild type p53 activity in HuH-7 (p53^Y220C^) similar to HuH-6 (p53^WT^). Cell viability assays in HuH-6 and HuH-7 cells revealed rather stronger cytotoxicity in HuH-7 cells (Fig. 7f) suggesting therapeutic potential of AL-βCD extracts for p53^Y220C^ harboring cancer cells.

## Discussion

Most of cancer cell types showed cytotoxicity to WiA and Wi-N, although to a variable extent, assigned to abrogation of mortalin-p53 complex resulting in nuclear translocation and reactivation of p53 activities ([5, 32–38] and data not shown). In our earlier study, we found that the i-Extract, possessing Wi-A and Wi-N as major ingredients, treated cancer cells with mutant p53 showed decrease in p53 expression suggesting enhanced destabilization and/or degradation (typical of wild type protein) [34]. In light of the fact that p53 mutations occur in a large variety of tumors as early event [9–12, 50, 51], we analysed the responses of main p53 hot spot mutations - 143A, 249S, 273C, 273H and 220C to Wi-A and Wi-N treatments. Computational analyses revealed a large difference in the interaction of withanolides with p53^WT^ and p53^Y220C^ and hence we performed extended experimental analysis to validate these predictions.

### Withanolides could selectively bind to p53^Y220C^ mutant and furnish wild type p53 function

Tyrosine 220 is known to play an important role in stabilization of β-sandwich region. The hydrophobic core maintained by its benzene ring gets disturbed when mutated with cysteine. This replacement leads to the solvation of binding cavity with the presence of more stable water molecules (Fig.3 b b) preventing the interactions of 220^th^ residue with the neighboring amino acids. Therefore, a small molecule that could prevent the solvation of cavity and maintain the hydrophobic nature of the core was anticipated to restore the wild type function in p53^Y220C^ protein.

Docking score of Wi-A, Wi-N, PhiKan7242 and PhiKan083 with p53^WT^ and p53^Y220C^ reflected their selective binding to the latter (Additional file 1: Table S4). PhiKans are known to restore the wild type activity to the mutant p53 by working as a bridge between the two loops (S3/4 and S7/S8), imparting them the stability similar to that observed in p53^WT^ [54, 55]. Similar to these PhiKan molecules, Wi-A was found to make hydrogen bonds with Thr 150 and Glu 221, residing in loop S3/4 and S7/S8 respectively. Further, Wi-A was surrounded by Val 147, Pro 151, Thr 155 from loop S3/4, and Cys 220, Glu 221, Pro 223 and Asp 228 from loop S7/8 of p53^Y220C^ (Fig. 4c and d). Wi-N also acquired the similar binding orientation (Fig. 4b). Wi-N was making four hydrogen bonds within the binding site - two with Thr 150, one with Gly 226 and Ser 227 each (Fig. 4e and f). Wi-N also made van der Waals contacts with Leu 145, Trp 146, Val 147, Asp148, Ser 149 and Pro 151 of loop S3/4 and Cys 220, Glu 221, Pro 222, Glu 224, Val 225 and Cys 229 of loop S7/S8. Wi-N thus also acted as a bridge between the two loops. Binding orientation of Wi-A and Wi-N was found to be complementary to the chemical nature of the binding site. The hydrophobic part of these molecules comprising of the steroid ring and a part of the lactone ring was completely buried inside the hydrophobic cavity (Fig. 4a and b), whereas the hydrophilic electronegative part including ketone, hydroxyl and epoxide groups was found interacting with either other residues of p53 or the solvent molecules.

Mortalin has been shown to inactivate p53 function by its cytoplasmic retention [4, 5, 31, 36–38]. Mortalin siRNA and small molecule drugs (MKT-077, CAPE and Artepillin) have been shown to abrogate mortalin-p53 interactions and reactivate growth arrest and apoptotic inducing functions of wild type and mutant p53 proteins [5, 31, 36–38, 60, 61]. Wi-A and Wi-N were also shown to abrogate mortalin-p53 interactions resulting in nuclear translocation and reactivation of tumor suppressor activity of wild type p53 [34, 36]. In light of this information and the current data, it is suggested that Wi-A and Wi-N may work by multiple mechanisms to activate p53 functions. These include (i) release of p53 from mortalin-p53 complexes in cancer cells, (ii) activation of nuclear translocation and reactivation of p53 activities including execution of DNA damage response, growth arrest and apoptosis and (iii) specific targeting of p53^Y220C^ and conformational reconstitution of its wild type functions. Wi-A and Wi-N, the biologically active withanolides with potent anticancer activity caused transcriptional activation function typical of wild type p53 in p53^Y220C^ cells inducing growth arrest and apoptosis. Taken together, we demonstrated that Wi-A and Wi-N are among the few small molecules that target p53^Y220C^ structural mutation and perhaps the first natural compounds with such activity. We demonstrated that Withanolide extract (enriched with Wi-A and Wi-N) possesses therapeutic potential and might be recruited for cancer treatment.

## Conclusion

Using molecular docking tools, we explored the possibility of selective targeting of mutant p53 proteins by Wi-A and Wi-N, and found that wild type and p53 mutant proteins p53^V143A^, p53^R249S^ and p53^R273H^ differ neither in structure/conformation nor in their binding to Wi-A/Wi-N. On the other hand, p53^Y220C^ showed conformational changes and strong interactions with Wi-N and Wi-A, resulting in reversion of its structural distortions and restoration of wild type tumor suppressor activity. We provide experimental evidence to such restoration of wild type p53 function in p53^Y220C^ mutant cells-treated with Wi-A/Withanolide extract. Withanolides, Wi-A and Wi-N, are therefore suggested as valuable natural drugs for treatment of cancers specifically carrying a p53^Y220C^ mutation.

## Additional file

Additional file 1: **Table S1.** PDB IDs of different p53 protein variants and their structural resolution. **Table S2.** H-bond network around residue 249 in p53^WT^ and p53^R249S^. **Table S3.** Binding score of Wi-A and Wi-N with different p53 mutants of DNA binding site. **Table S4.** Docking scores (XP docking) of withanolides and PhiKan with p53^WT^ and p53^Y220C^. **Figure S1.** Three domains of p53 protein. The structure of p53 contains N-terminal, central domain and C-terminal. **Figure S2.** (A) Immunostaining of control and Wi-A treated cells with anti-p53 antibodies detecting the total protein and mutant p53 specifically. Both antibodies detected the p53 protein in MRC-5hTERT p53^V143A^ and MRC-5hTert p53^Y220C^ showed decrease in p53 staining in the nucleus. Quantitation of total and mutant p53 from immunostaining images is shown in (B) and (C), respectively. **Figure S3.** (A) Immunostaining of control and doxorubicin (a DNA damage inducing reagent) treated cells with anti-53BP1 and p21^WAF-1^ antibodies. Increase in 53BP1 and p21^WAF-1^ was observed in treated cells. Quantitation of 53BP-1 and p21WAF1 from immunostaining images is shown in (B) and (C), respectively. **Figure S4.** Quantitation of immunostaining images of mortalin (A) and p53 (B) shown in Fig. 5; p21^WAF1^ and HP1g (shown in Fig. 6) and p53 (shown in Fig. 7). (PDF 8692 kb)

## Abbreviations

BCA: Bicinchonic Acid Assay
BSA: Bovine serum albumin
DBD: DNA binding domain
DMEM: Dulbecco’s modified Eagle’s medium
GAFF: General AMBER force field
JCRB: Japanese Collection of Research Bioresources
MD: Molecular dynamics
PAGE: Polyacrylamide gel electrophoresis
PME: Particle Mesh Ewald
PVDF: Polyvinylidene difluoride
Wi-A: Withaferin-A
Wi-N: Withanone
