# Supplementary material for: Wild type p53 function in p53Y220C mutant harboring cells by treatment with Ashwagandha derived anticancer withanolides: bioinformatics and experimental evidence
Source: J Exp Clin Cancer Res. 2019 Feb 26;38:103. doi: 10.1186/s13046-019-1099-x (PMC6390572; doi:10.1186/s13046-019-1099-x)

Wild type p53 function in p53^{Y220C} mutant harboring cells by treatment with Ashwagandha derived anticancer withanolides: Bioinformatics and experimental evidence

Durai Sundar[1] • Yue Yu[2] • Shashank P. Katiyar[1] • Jayarani Putri[2] • Jaspreet Kaur Dhanjal[1] • Jia Wang[2] • Anissa Nofita Sari[2] •E vangelos Kolettas[3] • Sunil C Kaul[2] • Renu Wadhwa[2]

Supplementary Materials

Table S1 PDB IDs of different p53 protein variants and their structural resolution.

**p53 type**	**PDB ID**	**Resolution (Å)**
$p53^{WT}$	3KMD	2.15
$p53^{V143A}$	2J1W	1.8
$p53^{Y220C}$	3ZME / 2J1X	1.35 / 1.65
$p53^{R249S}$	3D06	1.2
$p53^{R273C}$	4IBQ	1.8
$p53^{R273H}$	4IBS	1.78

Table S2 H-bond network around residue 249 in $p53^{WT}$ and $p53^{R249S}$.

H-bonds in $p53^{WT}$	H-bonds in $p53^{R249S}$
R249: E171	S249: Q167
R249: G245(BB)	S249: M246
R249: M246(BB)	
R249(BB): M246(BB)	
R248: N247	R248(BB): S240(BB)
R248(BB): S240(BB)	N247: G244(BB)
S240: V274(BB)	N247(BB): C242(BB)
S240(BB): V274(BB)	
Q165: H168	H168: Q167
Q165(BB): H168(BB)	H168(BB): E171(BB)
Q167: Q167(BB)	H168: Q167
R209:N208	R209: S207(BB)

BB → H-bond with Backbone

Table S3 Binding score of Wi-A and Wi-N with different p53 mutants of DNA binding site.

Molecule	$p53^{WT}/p53^{R249S}$ (kcal/mol)	$p53^{WT}/p53^{R273C}$ (kcal/mol)	$p53^{WT}/p53^{R273H}$ (kcal/mol)
Wi-A	-4.09 /-4.02	-3.82/-4.36	-3.82/-3.96
Wi-N	-3.71/-3.95	-4.21/-4.68	-4.21/-4.04

Table S4 Docking scores (XP docking) of withanolides and PhiKan with $p53^{WT}$ and $p53^{Y220C}$.

**Small molecule**	**Binding score with $p53^{WT}$ (kcal/mol)**	**Binding score with $p53^{Y220C}$ (kcal/mol)**	**Difference in binding score (kcal/mol)**
Wi-A	-1.68	-5.10	-3.42
Wi-N	-2.99	-7.29	-4.3
PhiKan7242	-2.28	-5.93	-3.65
PhiKan083	-2.03	-6.79	-4.76

Figure S1 Three domains of p53 protein. The structure of p53 contains N-terminal, central domain and C-terminal. The central core of p53 binds with the DNA via residues 112-141, 236-257, and 271-286. Mutations occurring in residues among 236-275 disrupt the interactions of p53 with DNA, whereas mutations in residues among 141-230 are involved in destabilization of p53.

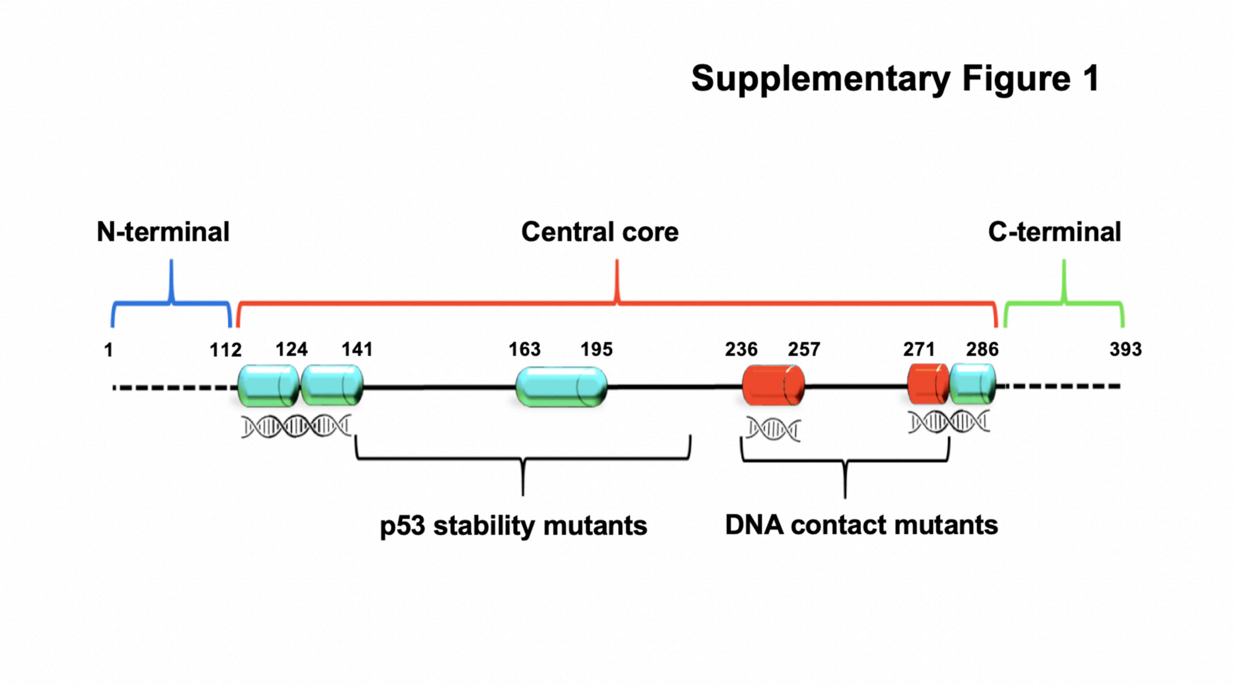

Figure S2. (A) Immunostaining of control and Wi-A treated cells with anti-p53 antibodies detecting the total protein and mutant p53 specifically. Both antibodies detected the p53 protein in MRC-5hTERT $p53^{V143A}$ and MRC-5hTert $p53^{Y220C}$ showed decrease in p53 staining in the nucleus. Quantitation of total and mutant p53 from immunostaining images is shown in (B) and (C), respectively.

Supplementary Figure 2

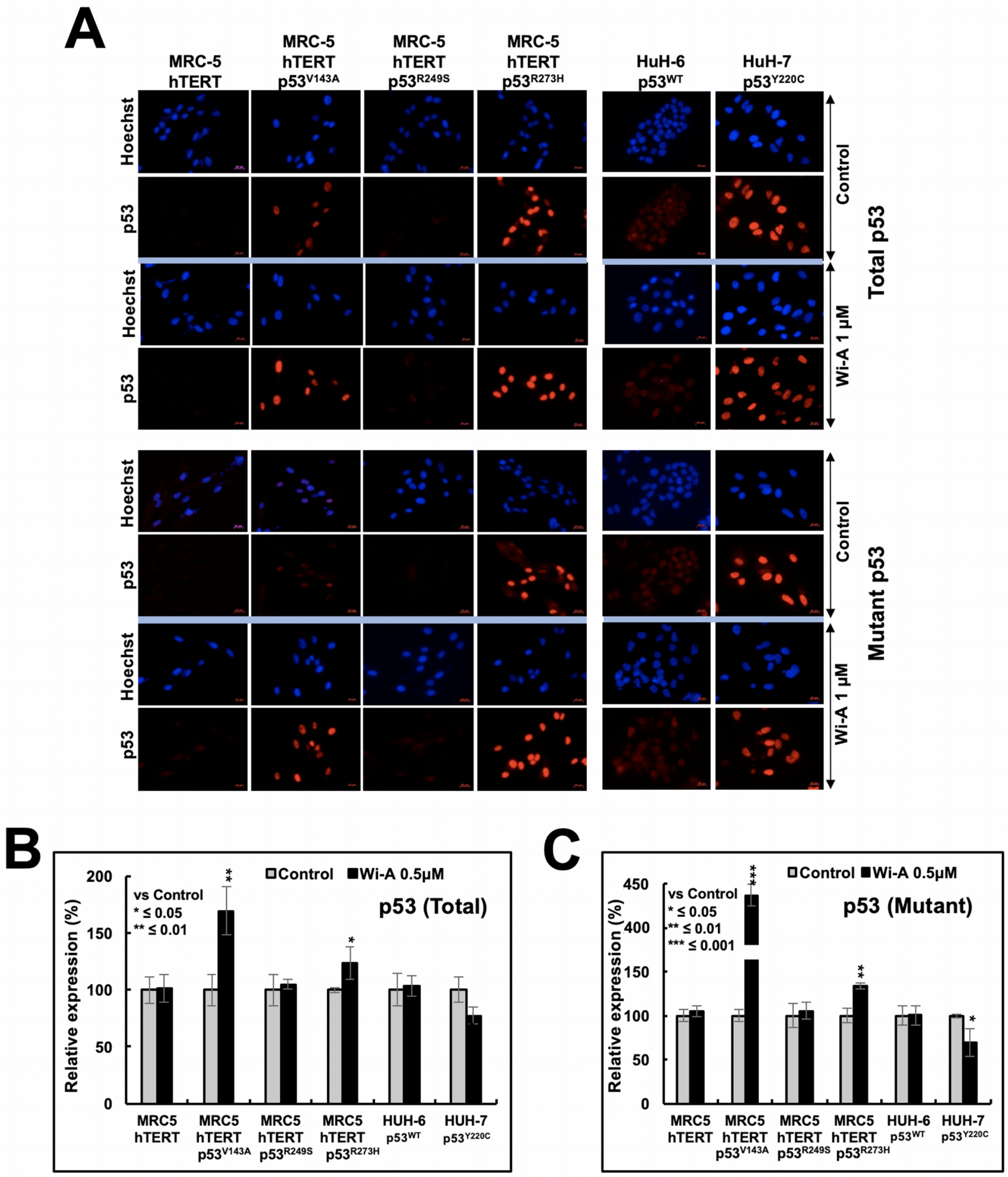

Figure S3 (A) Immunostaining of control and doxorubicin (a DNA damage inducing reagent) treated cells with anti-53BP1 and p21^{WAF-1} antibodies. Increase in 53BP1 and p21^{WAF-1} was observed in treated cells. Quantitation of 53BP-1 and p21^{WAF-1} from immunostaining images is shown in (B) and (C), respectively.

Supplementary Figure 3

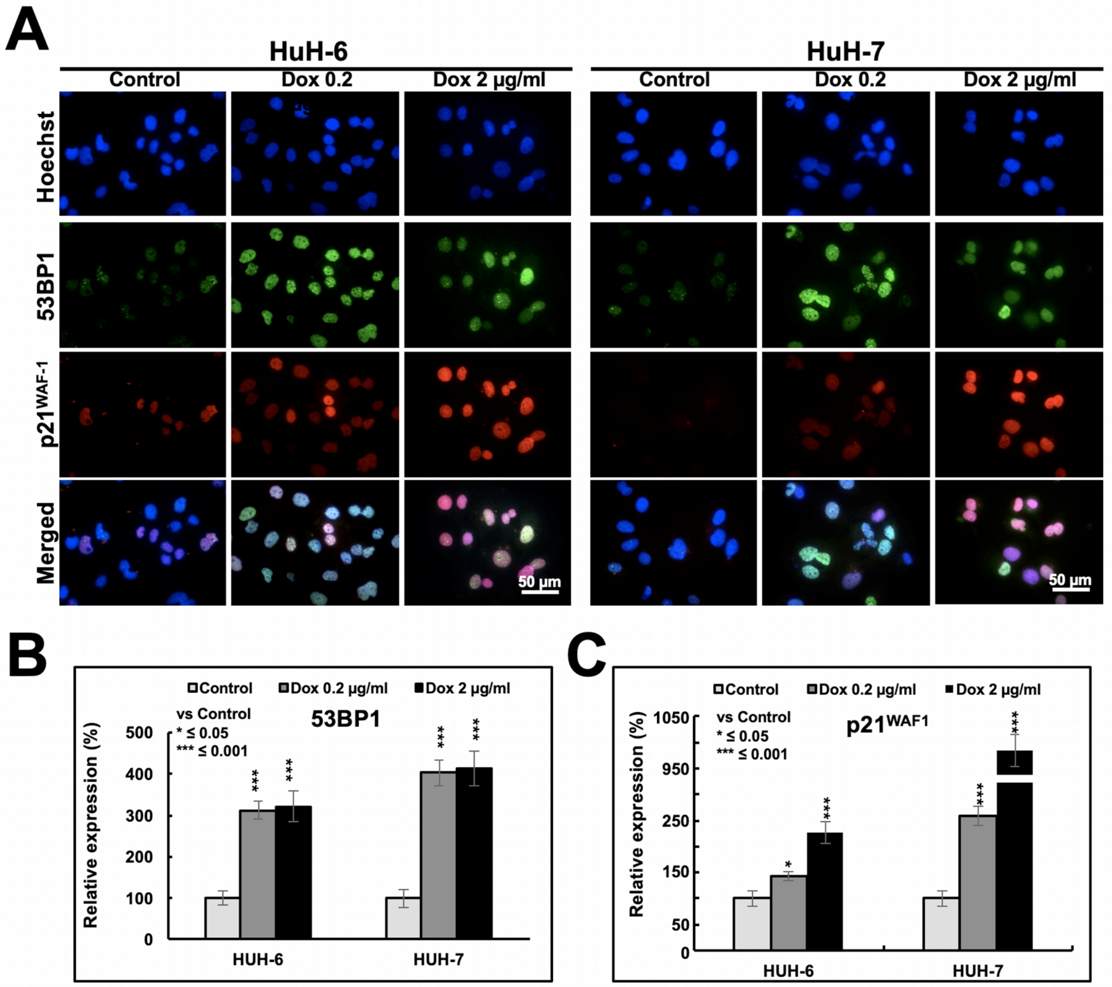

Figure S4 Quantitation of immunostaining images of mortalin (A) and p53 (B) shown in Fig. 5; p21^{WAF-1} and HP1γ (shown in Fig. 6) and p53 (shown in Fig. 7).

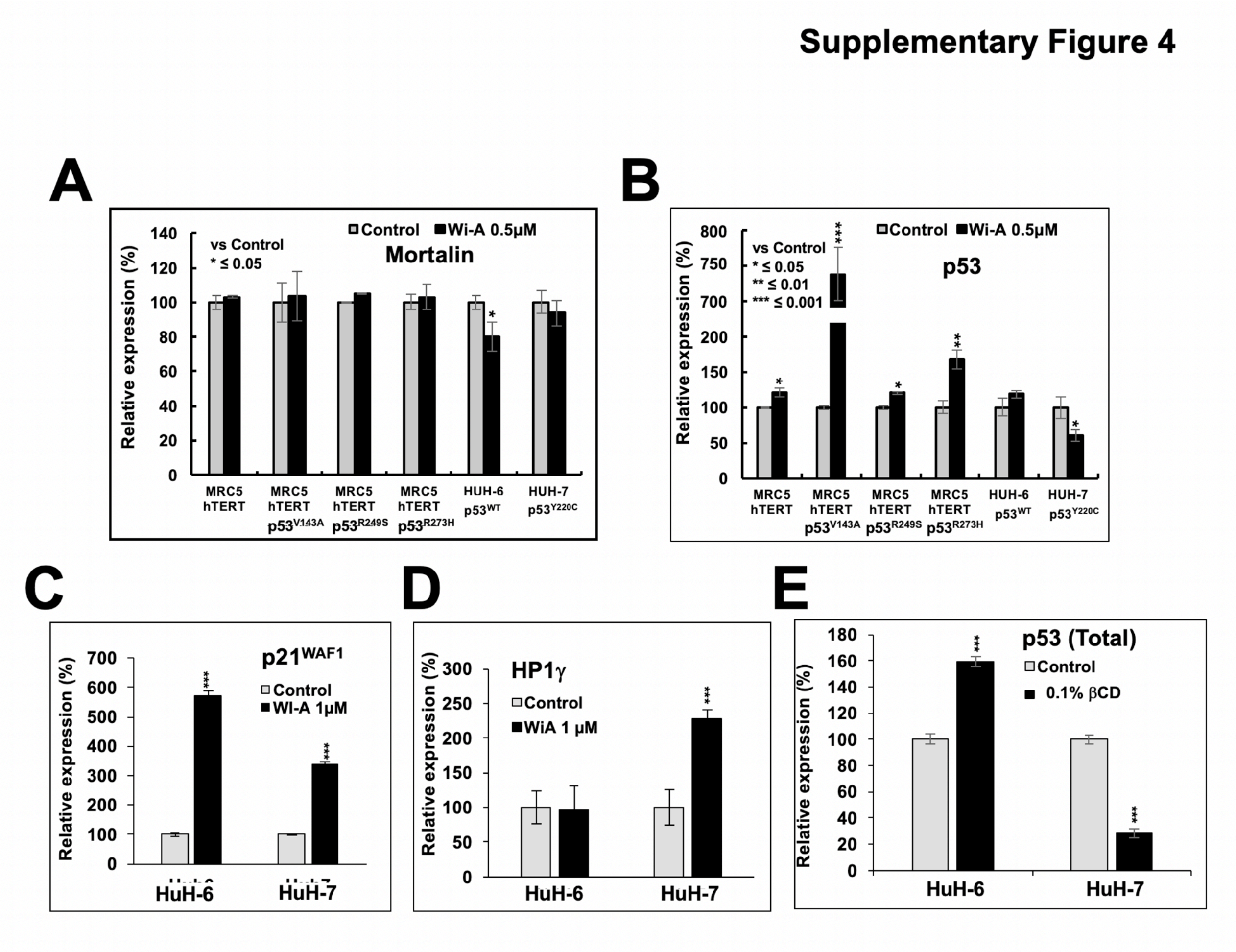

Supplement: Supplementary file 1 — Table S1. PDB IDs of different p53 protein variants and their structural resolution. Table S2. H-bond network around residue 249 in p53WT and p53R249S. Table S3. Binding score of Wi-A and Wi-N with different p53 mutants of DNA binding site. Table S4. Docking scores (XP docking) of withanolides and PhiKan with p53WT and p53Y220C. Figure S1. Three domains of p53 protein. The structure of p53 contains N-terminal, central domain and C-terminal. Figure S2. (A) Immunostaining of control and Wi-A treated cells with anti-p53 antibodies detecting the total protein and mutant p53 specifically. Both antibodies detected the p53 protein in MRC-5hTERT p53V143A and MRC-5hTert p53Y220C showed decrease in p53 staining in the nucleus. Quantitation of total and mutant p53 from immunostaining images is shown in (B) and (C), respectively. Figure S3. (A) Immunostaining of control and doxorubicin (a DNA damage inducing reagent) treated cells with anti-53BP1 and p21WAF-1 antibodies. Increase in 53BP1 and p21WAF-1 was observed in treated cells. Quantitation of 53BP-1 and p21WAF1 from immunostaining images is shown in (B) and (C), respectively. Figure S4. Quantitation of immunostaining images of mortalin (A) and p53 (B) shown in Fig. 5; p21WAF1 and HP1g (shown in Fig. 6) and p53 (shown in Fig. 7). (PDF 8692 kb) [file 13046_2019_1099_MOESM1_ESM.pdf]
